# *Brca2* deficiency drives gastrointestinal tumor formation and is selectively inhibited by mitomycin C

**DOI:** 10.1038/s41419-020-03013-8

**Published:** 2020-09-26

**Authors:** Xiaomin Chen, Fangfei Peng, Yan Ji, Honggang Xiang, Xiang Wang, Tingting Liu, Heng Wang, Yumin Han, Changxu Wang, Yongfeng Zhang, Xiangyin Kong, Jing-Yu Lang

**Affiliations:** 1 CAS_Key Laboratory of Tissue Microenvironment and Tumor, Shanghai Institute of Nutrition and Health, Shanghai Institutes for Biological Sciences, University of Chinese Academy of Sciences, Chinese Academy of Sciences, 200031 Shanghai, China; 2 Bioinformatics Core, Shanghai Institute of Nutrition and Health, Shanghai Institutes for Biological Sciences, Chinese Academy of Sciences, 200031 Shanghai, China; 3 Department of General Surgery, Pudong New Area People’s Hospital affiliated to Shanghai University of Medicine & Health Science, 201299 Shanghai, China

**Keywords:** Chemotherapy, Targeted therapies, Colon cancer, Gastric cancer

## Abstract

BRCA2 is crucial for repairing DNA double-strand breaks with high fidelity, and loss of BRCA2 increases the risks of developing breast and ovarian cancers. Herein, we show that *BRCA2* is inactively mutated in 10% of gastric and 7% of colorectal adenocarcinomas, and that this inactivation is significantly correlated with microsatellite instability. *Villin*-driven *Brca2* depletion promotes mouse gastrointestinal tumor formation when genome instability is increased. Whole-genome screening data showed that these *BRCA2* monoallelic and biallelic mutant tumors were selectively inhibited by mitomycin C. Mechanistically, mitomycin C provoked double-strand breaks in cancer cells that often recruit wild-type BRCA2 for repair; the failure to repair double-strand breaks caused cell-cycle arrest at the S phase and p53-mediated cell apoptosis of *BRCA2* monoallelic and biallelic mutant tumor cells. Our study unveils the role of BRCA2 loss in the development of gastrointestinal tumors and provides a potential therapeutic strategy to eliminate *BRCA2* monoallelic and biallelic mutant tumors through mitomycin C.

## Introduction

Homologous recombination (HR) is an evolutionarily conserved process for repairing DNA double-strand breaks (DSBs) with high fidelity^1^. Upon DNA damage, HR-repair proteins such as BRCA2 directly bind with RAD51 through BRC motifs and promote RAD51 filament formation to recruit homologous templates to complete error-free DSB repair^2–4^. The employment of BRCA1, which lacks physical interaction with RAD51 to damaged chromatin, is also important for HR-mediated DSBs but is not fully understood^5^. When BRCA1 and/or BRCA2 is defective, nonhomologous end-joining (NHEJ) with no requirement for homologous template is an alternative process used to repair DSBs with potential genome instability, which accelerates tumor development^6,7^.

*BRCA1* and *BRCA2* are frequently mutated at both the germline and somatic levels in human breast, ovarian, and pancreatic cancers^8,9^. For example, *BRCA2* is mutated in approximately 2.5% of breast cancers and 6% of ovarian cancers^10,11^. The inappropriate DSB repair caused by defective BRCA1/2 often leads to chromosomal dislocation, although this is not sufficient to drive tumor formation per se. However, simultaneous inactivation of *Trp53* strongly promotes spontaneous tumor formation in the breast, ovarian, and pancreatic organs of mice^12–15^. This suggests that *BRCA1/2* loss allows uncontrolled genetic mutations that drive tumorigenesis^16^.

Poly (ADP-ribose) polymerase (PARP) is a family of proteins that are responsible for repairing DNA single-strand breaks (SSBs). When *BRCA1* and *BRCA2* are fully depleted, PARP inhibition is synthetically lethal for these HR-defective tumors^17,18^. To date, PARP inhibitors have achieved remarkable clinical responses for treating BRCA1/2 deficient breast and ovarian tumors^19–21^. However, these HR-defective tumors quickly become resistant to PARP inhibitors as well as platinum analogues through secondary mutation of the *BRCA1/2* gene even in a single allele^22–24^. Therefore, the need for potent therapeutic agents to treat *BRCA1/2* monoallelic and biallelic mutant tumors is unmet.

Gastric and colorectal cancers are two major common cancer types worldwide, and the 5-year survival rates are ~30% and 60% for gastric and colorectal tumors, respectively^25,26^. To date, fluorouracil and platinum-based chemotherapy is still the first-line choice for treating gastrointestinal cancers, except for tumors with EGFR and HER2 expression^27^. Recently, the genetic landscapes of mutations in gastric and colorectal tumors have been well characterized and made known to the public^28–30^. By retrieving these data, we observed that *BRCA2* is mutated in gastric and colorectal tumor samples at frequencies of 10% and 7%, respectively, but the role of defective BRCA1/2 in gastrointestinal tumorigenesis is still unknown^5^. To address this question, we established a *Villin*-driven *Brca2* conditional knockout mouse to monitor gastrointestinal tumor formation, with or without the application of N-methyl-N-nitrosourea treatment or *Trp53* deletion. Meanwhile, we identified mitomycin C as the most effective agent to eliminate *BRCA2* monoallelic and biallelic mutant tumors by inhibiting BRCA2. Our study not only uncovers the role of *BRCA2* mutation in gastrointestinal tumorigenesis, but also provides a feasible therapeutic agent to potently eliminate *BRCA2* monoallelic and biallelic mutant tumors other than for those with p53 deficiency.

## Results

### *BRCA2* is frequently mutated in both gastric and colorectal tumor samples and is associated with microsatellite instability

**Fig. 1:**
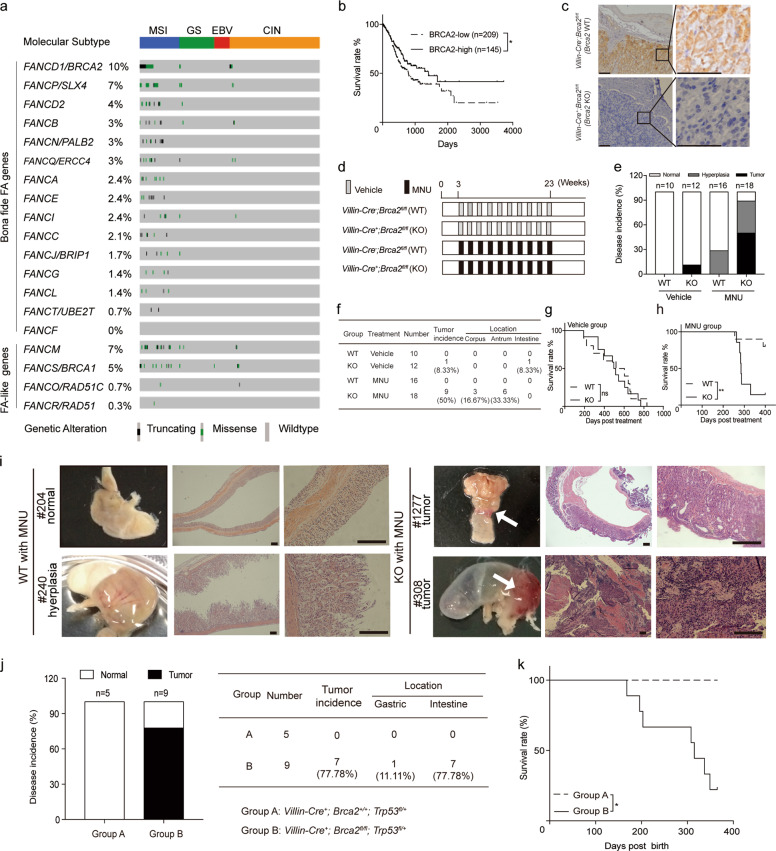
*Brca2* depletion in *Villin*-positive cells promotes gastrointestinal cancer formation by increasing genome instability. **a** Mutation status of FA- and FA-like members in 2014 TCGA stomach adenocarcinoma samples (*n* = 293). MSI, high microsatellite instability; GS, genomic stable; EBV, Epstein-Barr virus positivity; CIN, chromosomal instability. **b** Survival curves of BRCA2-low (*n* = 209) and BRCA2-high (*n* = 145) mRNA expression cases from provisional TCGA gastric cancer databases. *p < 0.05. **c** Brca2 staining in the stomach tissues of *Brca2* wild-type and knockout mice. Scale bar, 10 μm. **d** Experimental design: Three-week-old *Brca2* knockout mice or littermate wild-type mice were divided into four groups; MNU was administered to mice in two groups via drinking water at 120 mg/L on alternate weeks for a total exposure of 10 weeks, and vehicle was administered to the other two groups. **e** Disease incidence of *Villin-Cre*^*−*^*; Brca2*^*fl/fl*^ mice (*n* = 10, WT group), *Villin-Cre*^+^*; Brca2*^*fl/fl*^ (*n* = 12, KO group), *Villn-Cre*^−^*; Brca2*^*fl/fl*^ with MNU (*n* = 16, WT plus MNU group), and *Villin-Cre*^+^*; Brca2*^*fl/fl*^ with MNU (*n* = 18, KO plus MNU group). **f** Tumor incidence and location of all the tested mice in the four groups. **g** Kaplan–Meier survival curves of *Brca2* wild-type (*n* = 10) and knockout mice (*n* = 12) without MNU treatment. **h** Kaplan–Meler survival curves of *Brca2* wild-type (*n* = 8) and knockout mice (*n* = 6) with MNU treatment. **p < 0.01. **i** Gross morphology and H&E staining of stomach sections from 10-month-old MNU-treated *Brca2* wild-type and knockout mice. The white arrow indicates the visible tumors of *Brca2* knockout mice. Scale bar, 200 μm. **j** Disease incidence and location of *Villin-Cre*^+^*; Brca2*^+^^*/+*^*; Trp53*^*fl/+*^ mice (Group A, *n* = 5) and *Villin-Cre*^+^*; Brca2*^*fl/fl*^*; Trp53*^*fl/+*^ mice (Group B, *n* = 9). **k** Kaplan–Meier survival curves of *Villin-Cre*^+^*; Brca2*^+^^*/+*^*; Trp53*^*fl/+*^ and *Villin-Cre*^+^*; Brca2*^*fl/fl*^*; Trp53*^*fl/+*^ mice. *p < 0.05.

By retrieving the TCGA stomach cancer database^28,31,32^, we observed that genes belonging to the Fanconi anaemia (FA) pathway were frequently mutated in gastric adenocarcinoma^33–35^ (Fig. 1a). *BRCA2*, also termed *FANCD1*, ranked as the top candidate of 15 bona fide FA members at a frequency of 10% mutation (41/293), while *BRCA1*, another FA-like member, had an ~5% mutation rate (17/293). A similar pattern was also observed in colorectal cancer samples^30^, showing that *BRCA1* and *BRCA2* were mutated in ~4% (26/619) and 7% (59/619) of colorectal tumor samples, respectively (Fig. S1a). Thus, we selected the top candidate *BRCA2* to perform more studies. Notably, *BRCA2* mutations were associated with three features: (1) they were likely inactive (3 nonsense, 23 frameshift, and 74 missense mutations); (2) they were significantly associated with a microsatellite instability tumor subtype (*p* = 1.042E^−11^ in 293 gastric tumor samples and *p* = 7.39E^−11^ in 619 colorectal tumor samples by two-sided Fisher’s exact test), suggesting that frameshift mutations of *BRCA2* gene (20/23 cases) might be caused by deregulation of the mismatch repair pathway (Supplemental Table 1)^36^; and (3) they were not significantly associated with *TP53* mutations (*p* = 0.73 and *p* = 0.079 in gastric and colorectal tumors, respectively, by two-sided Fisher’s exact test). To examine the relationship between BRCA2 and patient survival, we further selected the TCGA provisional gastric and colorectal tumor databases to perform survival rate analysis, and the results showed that the low levels of BRCA2 mRNA expression were significantly correlated with the poor survival rates of both gastric and colorectal cancer patients (gastric: *p* = 0.04, *n* = 354; colon: *p* = 0.04, *n* = 597; Figs. 1b and S1b). Taken together, these data led us to assume that BRCA2 deficiency was pivotal for developing gastrointestinal tumors.

### *Villin*-driven *Brca2* deficiency induces tumorigenesis in gastrointestinal epithelial cells

To verify the role of *BRCA2* deficiency in tumor development, we generated *Villin-Cre*^*+*^*; Brca2*^*fl/fl*^ mice to achieve *Brca2* deletion in gastrointestinal epithelial cells^12,37^. *Brca2* was selectively depleted in gastrointestinal epithelial cells of *Brca2* knockout mice at protein and genetic levels compared to that of *Brca2* wild-type mice (Figs. 1c, S1c and S1d). When housed in specific pathogen-free conditions, *Villin-Cre*^*+*^*; Brca2*^*fl/fl*^ (KO) mice had a very small chance of developing spontaneous intestinal tumor (1/12 mice) compared with the *Villin-Cre*^*-*^*; Brca2*^*fl/fl*^ (WT) group (Fig. 1d–f), showing no difference in the median survival time between the two groups (505 vs 560.5 days, *p* > 0.05, Fig. 1g). These data suggest that *Brca2* deficiency alone is not sufficient to promote gastrointestinal tumorigenesis.

Gastrointestinal tumor-associated risk factors such as Helicobacter pylori infection, nitrate and nitrite uptake often lead to genome instability^38,39^. Therefore, we selected N-methyl-N-nitrosourea (MNU), an alkylating agent widely accepted for inducing gastrointestinal tumorigenesis^40^, to test whether *Brca2* deficiency can elicit gastrointestinal tumor formation when increasing genome instability (Fig. 1d). MNU treatment strongly provoked the formation of spontaneous and invasive gastric adenocarcinomas at both antrum and corpus sites in 9 out of 18 mice in the KO group (50%), and hyperplasia in the majority of the remaining mice (40%) (Fig. 1e, f, and i). Additionally, MNU-treated KO mice had a much lower survival rate than MNU-treat WT mice (*p* < 0.01, Fig. 1h). As a control, MNU-treated WT mice had no detectable tumors with normal and hyperplastic conditions observed in 70% and 30% of all tested mice, respectively (Fig. 1e, f, and i). These data suggest that *Brca2* deficiency can boost gastrointestinal tumor formation when genomic DNA is damaged.

p53 is a key guardian of genome stability that prevents cells from entering the S phase with unrepaired DNA sequences^41^. When heterozygously inactivated, it robustly facilitates breast tumor formation in *Brca2* conditional knockout mice^12^. Thus, we asked whether *Brca2* loss can promote gastrointestinal tumorigenesis when *Trp53* is heterozygously depleted by *Villin-Cre* expression (Fig. S1c and S1e). In the *Villin-Cre*^*+*^*; Brca2*^*fl/fl*^*; Trp53*^*fl/+*^ group (Group B), seven out of nine tested mice (78%) developed spontaneous and invasive adenocarcinomas at stomach and/or intestine sites, while no tumors detected in the *Villin-Cre*^*+*^*; Brca2*^*+/+*^*; Trp53*^*fl/+*^ group (Group A, 5/5 mice) (Fig. 1j). The H&E staining of tumor samples is shown in Fig. S1f. Mice in group B had a significantly lower survival rate than those in group A (*p* < 0.05, log-rank test, Fig. 1k).

Collectively, *Brca2* deficiency driven by *Villin*-Cre can boost gastrointestinal tumor formation when genome instability is increased. In particular, *Brca2* deficiency promotes gastric tumor formation when genome stability is selectively impaired in gastric epithelial cells.

### Mitomycin C potently eliminates *BRCA2* monoallelic and biallelic mutant tumor cells

**Fig. 2:**
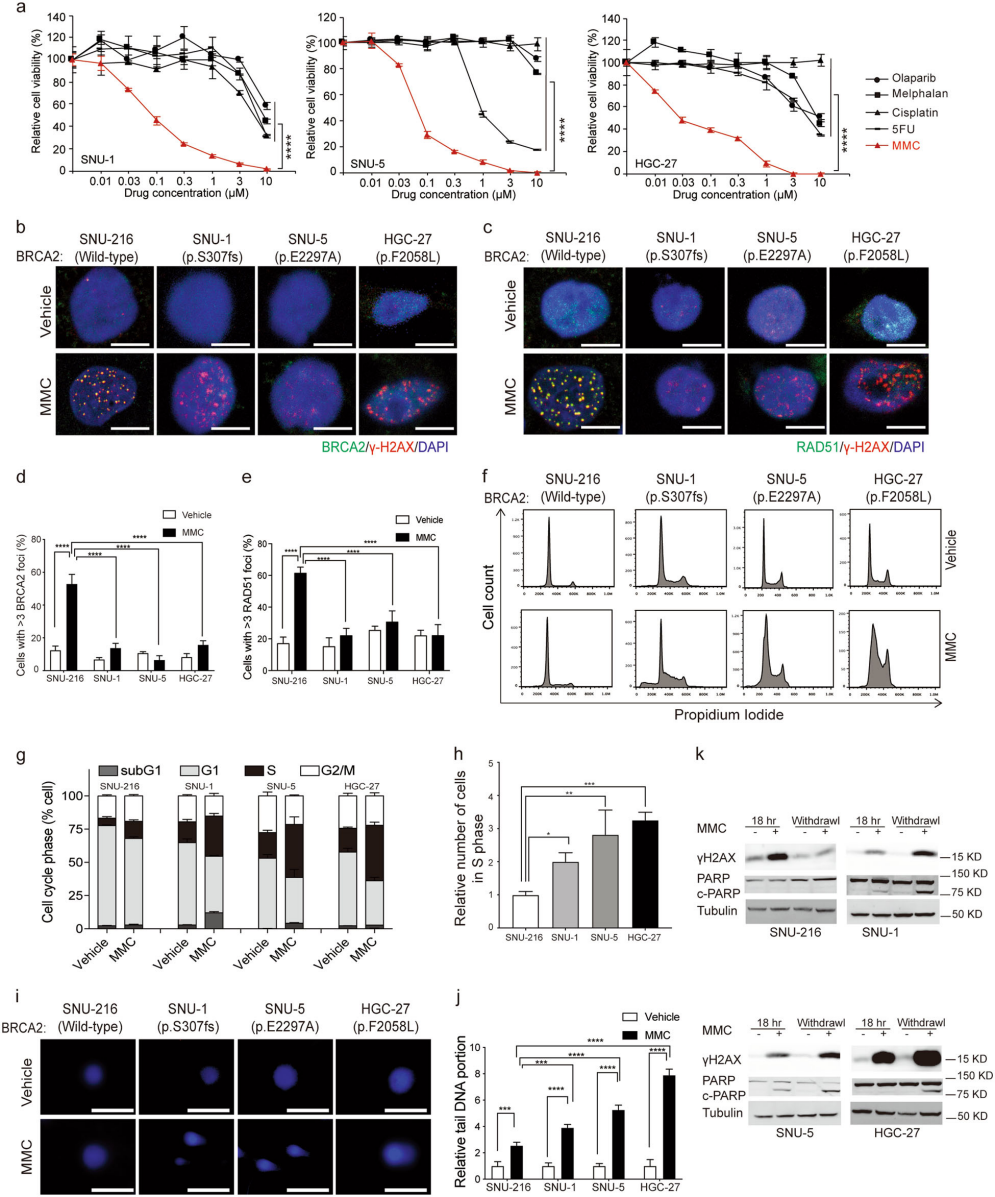
MMC induces DSBs and shows potent killing effects in *BRCA2* monoallelic and biallelic mutant tumor cells. **a** After SNU-1, SNU-5 and HGC-27 cells were treated with the indicated drugs for 72 h, the cell viability was determined at OD_570_, with normalization to DMSO treatment. **b** Representative images of γ-H2AX and BRCA2 foci formation in *BRCA2* wild-type and mutant cell lines after treated with 0.3 μM MMC for 18 h. Scale bar, 10 μm. **c** Representative images of γ-H2AX and RAD51 foci formation in *BRCA2* wild-type and mutant cell lines after treatment with 0.3 μM MMC or vehicle for 18 h. Scale bar, 10 μm. **d** The quantification of γ-H2AX and BRCA2 foci formation in *BRCA2* wild-type and mutant cell lines in the absence and presence of 0.3 μM MMC treatment for 18 h. **e** The quantification of γ-H2AX and RAD51 foci formation in *BRCA2* wild-type and mutant cell lines after treatment with 0.3 μM MMC or vehicle for 18 h. **f** The flow cytometry data of SNU-216, SNU-1, SNU-5, and HGC-27 after treatment with 0.1% DMSO or 1 μM MMC for 24 h. **g** The cell-cycle phases of SNU-216, SNU-1, SNU-5, and HGC-27 were presented after treatment with 0.1% DMSO or 1 μM MMC for 24 h. **h** The relative S phases of SNU-216, SNU-1, SNU-5, and HGC-27 were calculated and statistical analyzed after treatment with 0.1% DMSO or 1 μM MMC for 24 h. **i** Single-cell electrophoresis in the indicated cell lines after treatment with 3 μM MMC or vehicle for 36 h. Scale bar, 10 μm. **j** Quantification of single-cell electrophoresis in indicated cell lines. **k** Immunoblotting analysis of γ-H2AX, PARP and cleaved-PARP in the indicated cell lines after treatment with 0.3 μM MMC for 18 h plus withdrawal for 24 h. Data represent the mean ± SD of 3–5 replicates, *p < 0.05, **p < 0.01, ***p < 0.001, ****p < 0.0001.

In the literature, cancer cells with full BRCA2 deficiency are sensitive to DNA-damaging agents including mitomycin C (MMC)^6^, cisplatin^42^, melphalan^43,44^ and olaparib^17–21^. Therefore, we examined whether gastrointestinal cancer cell lines harboring *BRCA2* mutations are sensitive to these DNA-damaging agents. The great majority of gastrointestinal tumor cell lines used in this study harbor *BRCA1/2* heterozygous mutations; the exception is HGC-27, which harbors homozygous *BRCA2*^*p.F2058L*^ mutation (Fig. S2a)^45^. Cell viability data demonstrated that olaparib, MMC and melphalan significantly decreased the viability of *BRCA2* monoallelic and biallelic mutant gastrointestinal tumor cells at a concentration of 10 µM compared with that of *BRCA2* wild-type tumor cells (Fig. S2b–S2e). Consistent with previous reports^46^, 1 µM olaparib treatment for 5 days significantly decreased about 50% of the viability of HGC-27 cells (Fig. S2f). Cisplatin achieved significant inhibition in HGC-27 cells when exposed for a longer time at 20 µM (Fig. S2g). Among all the tested chemicals, MMC exhibited the most potent inhibitory effects in all six *BRCA2* monoallelic and biallelic mutant gastrointestinal cancer cell lines within 3 days at a concentration of 300 nM, surpassing the extent of inhibition by olaparib and other DNA-damaging agents (Fig. 2a). The *BRCA2* mutations of SNU-1, SNU-5, and HGC-27 cell lines were validated by Sanger sequencing, showing two monoallelic mutations and one biallelic mutation of the *BRCA2* gene in these three cell lines (Fig. S2h).

Because BRCA2 recruits RAD51 to DBS sites for executing HR repair^3^, we asked whether BRCA2/RAD51 signaling is impaired in these *BRCA2* monoallelic and biallelic mutant tumor cell lines. MMC treatment induces strong phosphorylation of histone H2AX at Ser 139 (γ-H2AX) (Fig. 2b, c, and k), which is a very early event of DNA damage responses that recruits DNA damage response proteins such as BRCA2 to DSB sites^47^. BRCA2 foci formation data showed that MMC provoked strong BRCA2 foci formation in the *BRCA2* wild-type cell line, which overlapped well with γ-H2AX staining, but it had little or no foci formation in all three *BRCA2* monoallelic and biallelic mutant gastrointestinal cancer cell lines (*p* < 0.0001, Fig. 2b and d). Consistently, RAD51 foci formation data revealed that MMC elicited strong RAD51 foci formation in *BRCA2* wild-type cells, showing colocalization with γ-H2AX, but that it failed to do so in *BRCA2* monoallelic and biallelic mutant cells (*p* < 0.0001, Fig. 2c and e). These data suggest that BRCA2/RAD51 signaling is functionally inactivated in *BRCA2* monoallelic and biallelic mutant cancer cells upon MMC treatment.

### MMC provokes irreversible DNA damage in *BRCA2* monoallelic and biallelic mutant cancer cells

MMC, an antibiotic obtained from *Streptomyces caespitosus*^48^, causes numerous DSBs in cells by selectively crosslinking GC-rich DNA due to CpG-specific two-step alkylation^49,50^. When DNA replication is stalled, cells arrest at the G1/S transition to preserve enough time to execute DNA repair; failure to repair severe DNA damage activates apoptotic signaling to eradicate these cells, preventing the rise of DNA mutations and genome instability^51^. Cell-cycle data showed that MMC markedly arrested these *BRCA2* monoallelic and biallelic mutant cell lines at the S phase after 24 h of treatment; in contrast, no significant cell arrest was observed in *BRCA2* wild-type cells (Fig. 2f, g, and h). DNA comet tail movement data showed that compared with *BRCA2* wild-type cells, severe DNA damage occurred in *BRCA2* monoallelic and biallelic mutant cells after MMC treatment for 36 h (Fig. 2i and j).

Interestingly, γ-H2AX was sustained at high levels in all three *BRCA2* monoallelic and biallelic mutant tumor cell lines even after MMC removal but returned to baseline phosphorylation status in wild-type SNU-216 cells during the same time frame (Fig. 2k). The irreversible DSB damage finally resulted in the apoptosis of three *BRCA2* monoallelic and biallelic mutant tumor cell lines but not of *BRCA2* wild-type cells (Fig. 2k). Based on these data, we conclude that MMC induces large amounts of DSBs in cells, which can be quickly removed by *BRCA2* wild-type cells, and that the failure to correct these DSBs in BRCA2-defective tumors leads to cell apoptosis.

### BRCA2 is a crucial therapeutic target of MMC

**Fig. 3:**
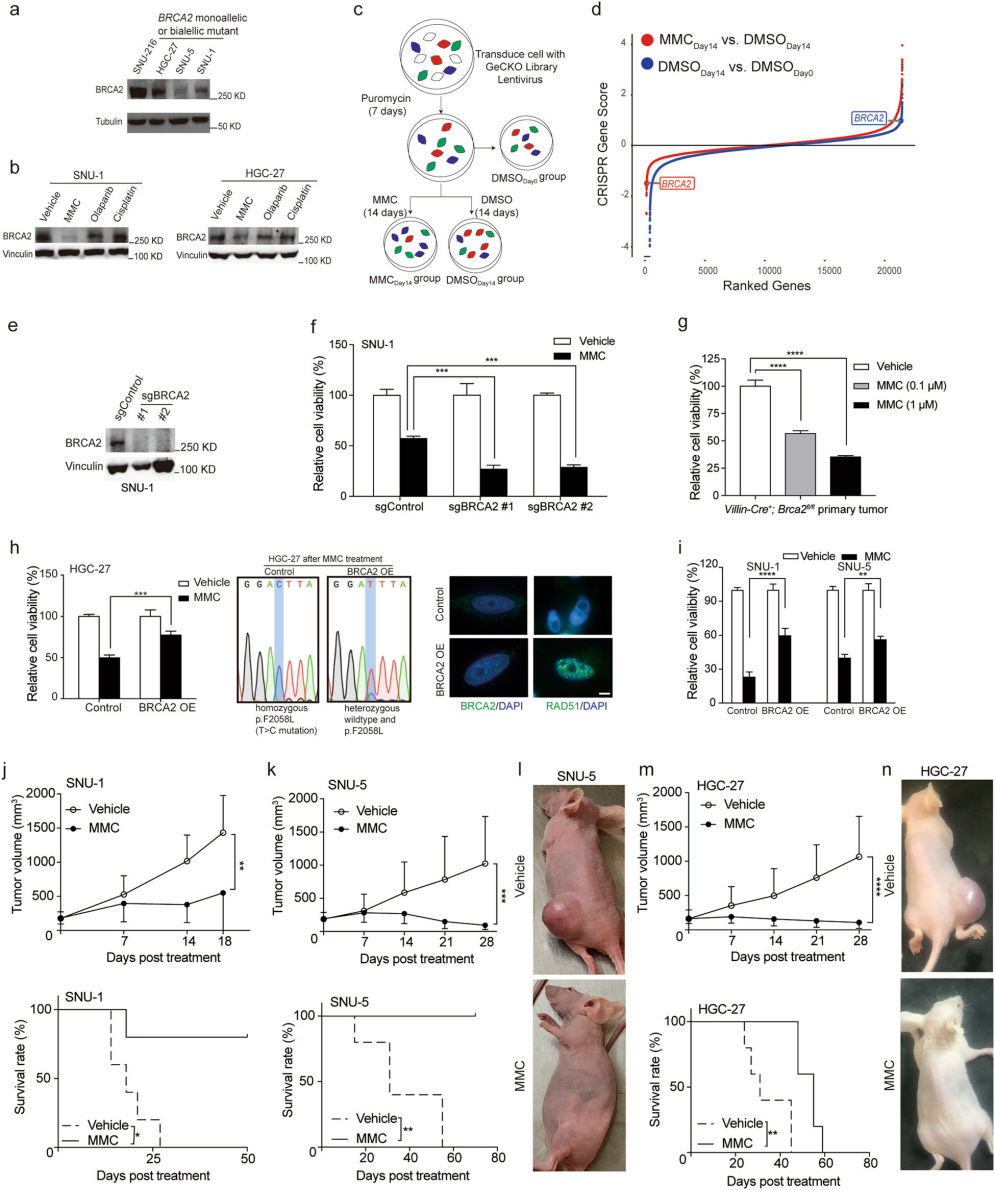
Whole-genome screening reveals that MMC eliminates *BRCA2*-mutant gastrointestinal tumor by targeting BRCA2. **a** The BRCA2 protein expression in *BRCA2* wild-type and mutant gastrointestinal tumor cell lines was shown by immunoblotting. **b** The BRCA2 protein expression was determined in SNU-1 and HGC-27 cell lines by immunoblotting after treatment with the indicated drugs. **c** Working flowchart of genome-scale CRISPR-Cas9 knockout (GeCKO) screening. **d** CRISPR score analysis data showed that *BRCA2* knockout selectively sensitized SNU-1 cells to MMC treatment, but did not affect the viability of SNU-1 cells when treated with vehicle. **e** The knockout efficiency of BRCA2 protein was determined by immunoblotting. **f** sgBRCA2 and sgControl SNU-1 cells were treated with 0.1 μM MMC or 0.1% DMSO for 72 h, respectively, and the cell viability was determined at OD_570_. **g** Primary tumor cells derived from tumor tissues of *Villin-Cre*^+^*; Brca2*^*fl/fl*^ mice (#1305) were treated with 0.1 and 1 μM MMC or 0.1% DMSO for 72 h. **h** Cell viability, expression status of BRCA2 and foci formation in HGC-27 cells treated with MMC after introduction with wild-type BRCA2 or control vector. Scale bar, 10 μm. **i** The viability of SNU-1 and SNU-5 cells in the absence or presence of wild-type BRCA2 was determined when treatment with 0.1 μM MMC for 72 h, 0.1% DMSO was used as a control. **j** Tumor volume and survival rate of *Rag2*^*−/−*^*;Il2r*^*−/*−^ mice harboring SNU-1 tumor xenografts. Mice were intraperitoneally treated with vehicle or 1 mg/kg MMC, once per week for total four weeks, *n* = 5. **k** Tumor volume and survival rate of nude mice with SNU-5 tumor xenografts. Mice were intraperitoneally treated with vehicle or 1 mg/kg MMC, once per week for total four weeks, *n* = 5. **l** Representative images of SNU-5 tumor-bearing nude mice after 3 weeks of vehicle or MMC treatment. **m** Tumor volume and survival rate of nude mice with HGC-27 tumor xenografts. Mice were intraperitoneally treated with vehicle or 1 mg/kg MMC, once per week for total 4 weeks, *n* = 5. **n** Representative images of HGC-27 tumor-bearing nude mice after three weeks of vehicle or MMC treatment. Data represent the mean ± SD, *p < 0.05, **p < 0.01, ***p < 0.01, ****p < 0.0001.

Next, we asked why *BRCA2* monoallelic and biallelic mutant gastrointestinal tumor cells were hypersensitive to MMC. As shown in Fig. 3a, BRCA2 protein was expressed at lower levels in monoallelic and biallelic mutant tumor cell lines than in wild-type cell lines. Importantly, MMC significantly decreased BRCA2 expression at both the protein and mRNA levels in *BRCA2* monoallelic and biallelic mutant cell lines but not in *BRCA2* wild-type cell lines (Figs. 3b, S3a, S3b, and S3c)^52–54^. Olaparib and cisplatin had minor inhibitory effects on BRCA2 protein expression levels. These data suggest that BRCA2 inhibition is critical for achieving the therapeutic effects of MMC, which explains why MMC has better antitumor activity than other DNA-damaging reagents due to the inhibition of BRCA2 expression.

To verify this hypothesis, we applied genome-scale CRISPR-Cas9 knockout (GeCKO) screening to simultaneously knockout 19050 human genes in SNU-1 cells to explore whether *BRCA2* monoallelic mutant tumor cells are selectively targeted by MMC^55^. The experimental procedure of GeCKO screening is shown in Fig. 3c and the Material and Methods^56^. At the endpoint of treatment, at least 2 × 10^7^ cells in each group were harvested to achieve 300-fold coverage of the sgRNA library. After analysing the sequencing data with a MAGeCK algorithm^57^, we obtained a total of 18 gene candidates that significantly enhanced the MMC killing effects but did not reduce the cell viability of the control group when they were removed (ratio < 0.5, FDR < 0.05, with duplicated hits per screening, Supplemental Table 2). *BRCA2* was the top 1 candidate selected in this screening (ratio = 0.3, FDR < 0.001, Fig. 3d). By contrast, *BRCA1* showed no significant change during the screening (ratio = 1.68, *p* = 0.12), probably due to the inability to physically associate with RAD51^5^. In addition, we performed a new round of GeCKO screening in *BRCA2* wild-type SNU-216 cells, showing that FA genes, including *FANCB, FANCF, FANCM*, and *FANCD2*, were positively selected (Fig. S3d). This suggests that knockout of FA genes upstream of *BRCA2* has minor effects when *BRCA2* is largely impaired but that these genes are functionally important when *BRCA2* is intact. Nevertheless, whole-genome screening data suggest that BRCA2 inhibition is critical for optimizing the therapeutic effects of MMC.

Next, we fully depleted BRCA2 protein expression in both alleles of SNU-1 cells using two different sgRNAs (Figs. 3e and S2i). As expected, these full BRCA2-deficient cells were more sensitive to MMC than sgControl cells (*p* < 0.001, Fig. 3f), suggesting that BRCA2 function was impaired but was still active in SNU-1 cells. To support our notion, primary tumor cells derived from MNU-treated *Villin-Cre*^*+*^*; Brca2*^*fl/fl*^ murine spontaneous tumors were dose-dependently inhibited by MMC (*p* < 0.0001, Fig. 3g). HGC-27 cells were less sensitive to MMC when wild-type BRCA2 was re-expressed (*p* < 0.001), as evidenced by the fact that homozygous *BRCA2*^*p.F2058L*^ alleles were primarily replaced by the wild-type *BRCA2* allele (Fig. 3h). MMC treatment could induce the formation of BRCA2 and RAD51 foci when BRCA2 was reintroduced into HGC-27 cells but not in the control cells, indicating that the HR-repair ability was partially restored in the BRCA2-re-expressing cells (Fig. 3h). Similarly, both SNU-1 and SNU-5 cells were more insensitive to MMC when wild-type BRCA2 was reintroduced (Fig. 3i). Taken together, we conclude that BRCA2 is a target that could be selectively inhibited by MMC.

### MMC eliminates *BRCA2* monoallelic and biallelic mutant tumor xenografts in vivo

Next, we tested the in vivo efficacy of MMC in three *BRCA2* monoallelic and biallelic mutant tumor xenograft models. Compared to vehicle treatment, MMC treatment potently inhibited the in vivo growth of heterozygous *BRCA2* monoallelic mutant SNU-1 tumor xenografts in immune-deficient mice (*p* < 0.01, Fig. 3j top) and significantly improved the survival rate of the mice (*p* < 0.05, Fig. 3j bottom). Similar results were obtained in two other *BRCA2* monoallelic and biallelic mutant tumor xenograft models (*p* < 0.01, Fig. 3k and m upper), and significantly improved survival rates for the MMC-treated mice compared with the control mice (*p* < 0.01, Fig. 3k and m bottom). Notably, MMC induced complete tumor regression in three out of five mice bearing HGC-27 tumor xenografts and five out of five mice bearing SNU-5 tumor xenografts (Fig. 3l and n).

### p53 is responsible for MMC-induced apoptosis of *BRCA2* monoallelic and biallelic mutant gastrointestinal tumors

**Fig. 4:**
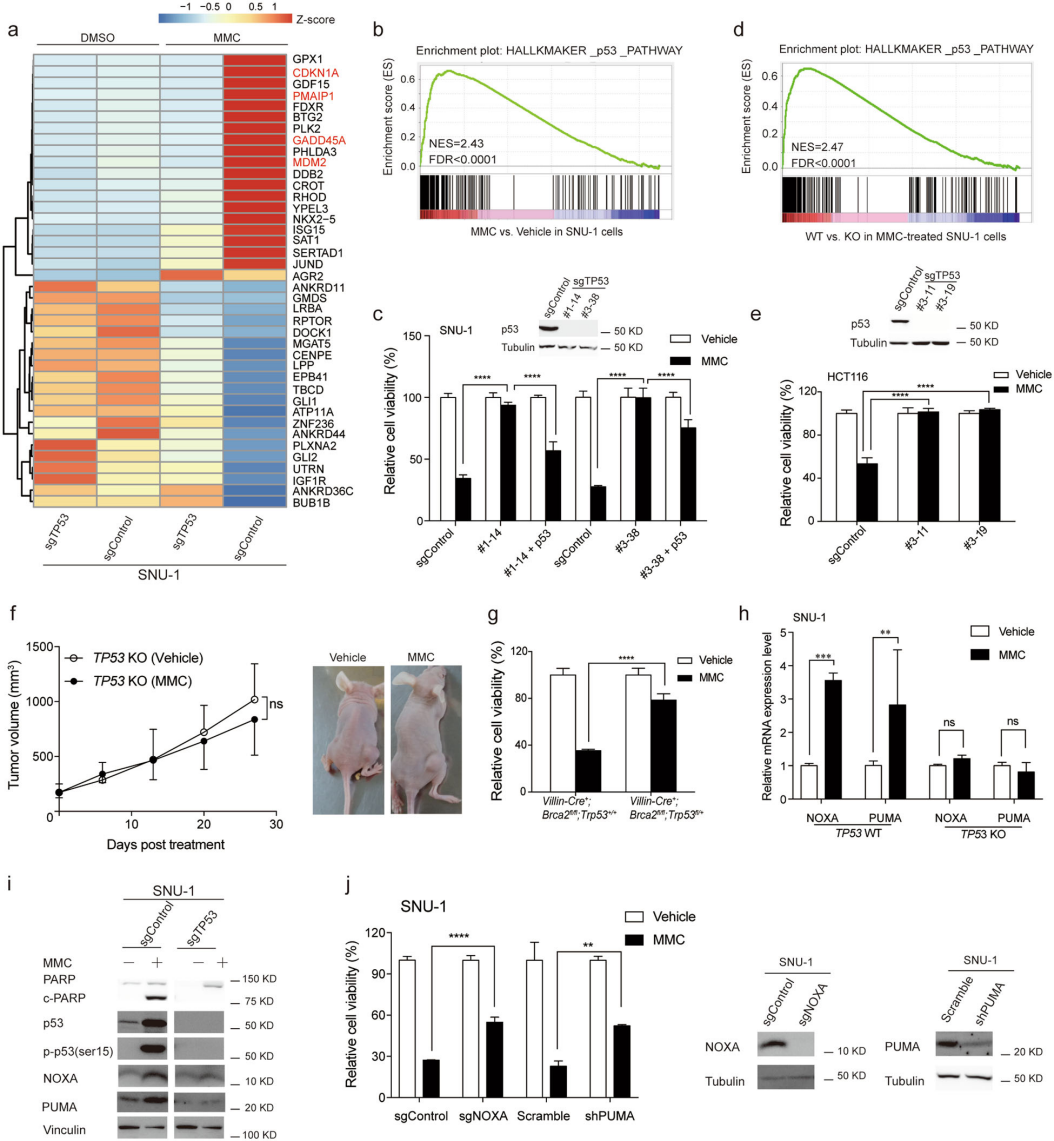
p53 is responsible for MMC-induced apoptosis of *BRCA2* monoallelic and biallelic mutant cells. **a** Nonbiased clustering of the top 20 up and downregulated genes by comparison MMC-treated to DMSO-treated SNU-1 cells. **b** Gene set enrichment analysis of the gene signature of the p53 pathway in SNU-1 cells treated with 3 μM MMC or 0.1% DMSO for 36 h. **c** SNU-1 sgTP53, sgControl and p53-putback cells were treated with 0.3 μM MMC or 0.1% DMSO for 72 h, and cell viability was determined at OD_570_. The knockout effect of p53 protein is shown in the upper panel. **d** Gene set enrichment analysis of the gene signature of the p53 pathway in SNU-1 (*TP53* WT) and SNU-1(*TP53* KO) cells treated with 3 μM MMC for 36 h. **e** HCT116 sgTP53 and sgControl cells were treated with 0.1 μM MMC or 0.1% DMSO for 72 h, and the cell viability was determined at OD_570_. The knockout effects of p53 protein are shown in the upper panel. **f** Tumor volume of SNU-1 (*TP53* KO) tumor xenografts in nude mice. Mice were intraperitoneally treated with vehicle or 3 mg/kg MMC, once per week for a total of four weeks, *n* = 5. Representative images are shown on the right. **g** Cell viability of primary cancer cells derived from *Villin-Cre*^+^*; Brca2*^*fl/fl*^*; Trp53*^+^^*/+*^ mouse or *Villin-Cre*^+^*; Brca2*^*fl/fl*^*; Trp53*^*fl/+*^ mice treated with 1 μM MMC or 0.1% DMSO for 72 h. **h** RT-qPCR analysis of NOXA and PUMA mRNA levels in SNU-1 sgControl and sgTP53 cells when treated with 3 μM MMC or 0.1% DMSO for 24 h. **i** Immunoblotting analysis of PARP, cleaved-PARP, p53, p-p53(ser15), NOXA and PUMA of sgControl and sgTP53 SNU-1 cells. Cells were treated with 3 μM MMC or 0.1% DMSO for 36 h. **j** Cell viability of the SNU-1 NOXA knockout cell line, control cell line (sgControl), SNU-1 PUMA KD cell line and control cell line (Scramble) treated with 0.1 μM MMC or 0.1% DMSO for 72 h. Cell viability was determined at OD_570_. The knockout effects of NOXA and knockdown effects of PUMA are shown on the right. Data are presented as the mean ± SD of 3–5 replicates, **p < 0.01, ***p < 0.001, ****p < 0.0001.

We further investigated how MMC triggers the apoptosis of *BRCA2* monoallelic and biallelic mutant cancer cells. Gene set enrichment analysis (GSEA)^58^ revealed that the gene signature of the p53 pathway was positively selected after comparing MMC with vehicle in SNU-1 cells (FDR < 0.0001, Fig. 4a and b). The mRNA expression levels of p53 transcriptionally regulated *CDKN1A*, *PMAIP1*, *MDM2*, and *GADD45A* genes were markedly increased after MMC treatment (Fig. 4a). Therefore, we asked whether p53 is responsible for MMC-induced cell apoptosis. To test this hypothesis, we depleted *TP53* gene expression in SNU-1 cells using two independent sgRNAs and observed that *TP53* knockout subclones were no longer sensitive to MMC compared with sgControl subclones (*p* < 0.0001, Fig. 4c). The p53 gene expression signature was also reversed by comparing MMC-treated sgControl and sgTP53 cells (FDR < 0.0001, Fig. 4d). The knockout effect of p53 protein in two subclones is presented in the upper panel of Fig. 4c. When wild-type p53 was reintroduced, these sgTP53 cells were re-sensitized to MMC (*p* < 0.0001, Fig. 4c), reflecting that it is not an off-target effect. Similar results were also observed in the *BRCA2*^*p.I2672fs*^ mutant HCT116 colorectal cancer cell line (*p* < 0.0001, Fig. 4e). Importantly, *TP53* knockout not only abolished the killing effects of MMC in vitro but also greatly reduced the therapeutic efficacy of MMC in vivo (*p* > 0.05, Fig. 4f). Primary cells derived from *Villin-Cre*^*+*^*; Brca2*^*fl/fl*^*; Trp53*^*fl/+*^ spontaneous tumor also became less sensitive to MMC than those derived from *Villin*-Cre^+^; *Brca2*^*fl/fl*^*; Trp53*^+/+^ (Fig. 4g).

Next, we asked which downstream gene is responsible for p53-mediated cell apoptosis. NOXA and PUMA are considered two key effectors of the p53 apoptosis pathway^59^. In this study, we observed that MMC significantly induced the mRNA and protein expression levels of both NOXA (encoded by the *PMAIP1* gene) and PUMA, but it was diminished when *TP53* was knocked out (Fig. 4h and i), suggesting that it is a p53-dependent event. Notably, individual inhibition of NOXA or PUMA significantly reduced the in vitro efficacy of MMC (both *p* < 0.01, Fig. 4j), indicating that NOXA and PUMA are required for p53-mediated apoptosis of *BRCA2* monoallelic and biallelic mutant cells upon MMC treatment. As we previously mentioned, *TP53* mutations were almost equally distributed in both *BRCA2* wild-type and mutant gastrointestinal tumors, and it would be of value to examine the genetic mutations of the *TP53* gene before applying MMC to patients with *BRCA2* monoallelic and biallelic mutant tumors.

## Discussion

It has been known for many years that BRCA2 deficiency increases the lifetime risk of developing breast, ovarian, and pancreatic cancers^60–63^. However, the role of *BRCA2* mutants in gastrointestinal cancer is still largely unknown. In this study, we first showed that *BRCA2* is mutated in ~7–10% of gastrointestinal tumors, and that it was significantly correlated with microsatellite instability. For example, 20 out of 23 frameshift mutations occurred at the polytract sites of the *BRCA2* gene. Coincidentally, a recent study reported that the tumors of 2.3% of gastrointestinal cancer patients harboring deleterious alterations in *BRCA2* were associated with high microsatellite instability^64^. Thus, it is a unique way for gastrointestinal tumors to develop *BRCA2* mutations that is distinct from how these mutations occur in breast and ovarian cancer.

Second, we showed that *Brca2* deficiency alone driven by *Villin*-*Cre* was not sufficient to promote gastrointestinal tumor formation but that it robustly elicited tumor formation when genome instability was increased. MNU treatment preferentially induced tumor in the stomach, while heterozygous depletion of *Trp53* provoked cancer in both stomach and intestine. In addition, we did not observe tumor formation when *Trp53* was heterozygously depleted alone in gastrointestinal epithelial cells. These data indicate that co-mutation of *Brca2* and *Trp53* may have a synergic effect towards inducing tumorigenesis.

BRCA2 deficiency can increase chromosomal instability when the error-free HR-repair system is impaired and eventually contributes to carcinogenesis^6^. p53 is a guardian of the genome for keeping the genome intact, and will prevent cells from entering the cell cycle with unrepaired DSBs^65^. When p53 is defective, *BRCA2*-mutant cells can quickly accumulate genomic instability and lead to tumorigenesis without inducing cell apoptosis. This may explain why other researchers cannot observe tumor formation when *Brca2* was deleted alone in the small intestine^66^. At the same time, we observed that MMC treatment induced cell apoptosis in a p53-dependent manner. Thus, we conclude that deregulated p53 can efficiently facilitate the formation of *BRCA2*-mutant gastrointestinal cancer.

In agreement with previous reports^6,43,44^, our genome-wide knockout screen data revealed that full depletion of *BRCA2* is critical for DNA-damaging agents to achieve the best therapeutic responses. For example, PARP inhibitors only kill the *BRCA2* fully inactivated tumor cells^17,18^. Interestingly, we found that MMC was remarkably effective for eliminating *BRCA2* monoallelic and biallelic mutant tumors by reducing BRCA2 mRNA and protein expression levels. The efficacy of MMC surpasses that of other DNA-damaging agents including cisplatin and olaparib. Thus, MMC offers a new therapeutic opportunity for patients harboring *BRCA2* monoallelic and biallelic mutations when they do not respond to PARP inhibitors.

## Materials and methods

### Cell culture

The human gastric cancer cell lines were purchased from KCLB, RCB, and JCRB cell banks. RKO, HCT116 cell lines were purchased from the cell bank of Shanghai Institutes for Biological Sciences, Chinese Academy of Sciences. HCT-15 was kindly provided by Dr. Zehong Miao, Shanghai Institute of Material Medica, Chinese Academy of Sciences. Cells were cultured in RPMI 1640, DMEM/F-12 or MEM medium with 10% fetal bovine serum and penicillin and streptomycin at 37 °C with 5% CO_2_. All the cell lines were recently authenticated by STR, keeping mycoplasma free.

### Mice genotyping and spontaneous tumor models

All the animal experiments were performed according to the procedures approved by the Institutional Animal Care and Use Committee, Shanghai Institute of Nutrition and Health. The *Brca2*^*fl/fl*^ mouse (01XB9, STOCK *Brca2*^*tm1Brn*^/Nci) and *Trp53*^*fl/fl*^ mouse (01XC2, FVB.129P2-*Trp53*^*tm1Brn*^/Nci) were the kind gifts from Drs. Jos Jonkers and Anton Berns with signed material transfer agreements from NIH^12^. *Villin-Cre* mouse (T002376, B6/JNju-Tg(Pvillin-cre)I/Nju) was purchased from NRCMM (Nanjing, China). BALB/c mice were purchased from Shanghai SLAC Laboratory Animal Co. Ltd. To obtain *Brca2* conditional knockout BALB/c mice in gastrointestinal epithelial cells, *Brca2*^*fl/fl*^ mouse was first crossed with *Villin*-*Cre* mouse, and then the offspring were backcrossed onto BALB/c background for at least six generations before the studies. To get double knockout mice of *Brca2* and *Trp53*, the *Villin*-*Cre*^+^; *Brca2*^*fl/fl*^ mouse was crossed with *Trp53*^*fl/fl*^ mouse and the offspring were backcrossed onto C57BL/6 background for at least three generations.

Mice were marked and genotyped about 10 days after born. Briefly, tail tip was boiled for 20 min in 200 µL of 0.05 M NaOH, then neutralized with 20 µL Tris-EDTA buffer (Tris 1 M, pH 8, EDTA 10 mM), serving as PCR template. The genotype of mouse was validated by the size of PCR products. For the *loxP* site in intron 10 of *Brca2*^*fl/fl*^ allele, the PCR products were 376 bp for the floxed allele and 298 bp for the wild-type allele, respectively. For the *loxP* site in intron 11 of *Brca2*^*fl/fl*^ allele, the PCR products were 529 bp for the floxed allele and 450 bp for the wild-type allele, respectively. Detection of the *Brca2*^Δ11^ allele yielded a 324-bp fragment. For the *loxP* site in intron 1 of *Trp53*^*fl/fl*^ allele, the PCR products were 370 bp for the floxed allele and 288 bp for the wild-type allele, respectively. For the *loxP* site in intron 10 of *Tp53*^*fl/fl*^ allele, the PCR products were 584 bp for the floxed allele and 431 bp for the wild-type allele, respectively. Detection of the of the *Trp53*^Δ2–10^ allele yielded a 612-bp fragment. Detection of the *Cre* yielded a 350-bp fragment. Primers for genotyping were listed in Supplementary Table 3.

For *Brca2* knockout experiment, mice were separated into four groups with indicated numbers after genotyped, termed as *Villin*-*Cre*^−^; *Brca2*^*fl/fl*^ mice without MNU, *Villin*-*Cre*^*+*^; *Brca2*^*fl/fl*^ without MNU, *Villin*-*Cre*^−^; *Brca2*^*fl/fl*^ with MNU and *Villin*-*Cre*^*+*^; *Brca2*^*fl/fl*^ with MNU. MNU was added in drinking water at 120 mg/L on alternate weeks for a total exposure of 10 weeks. The drinking water was freshly prepared every three days in light-shielded bottles. For *Brca2* and *Trp53* double knockout experiment, mice were divided into two groups with indicated numbers.

### Genome-scale CRISPR-Cas9 knockout screening

Lentivirus containing human GeCKOv2 library were produced by HEK293T and were precipitated by PEG buffer with a final concentration of 5% PEG8000 and 0.15 M NaCl. 2 × 10^8^ SNU-1 cells were spin-infected at 37 °C with virus containing library sgRNAs at a MOI of 0.3. After spininfection, cells were selected with 2 µg/ml puromycin for another 7 days. After puromycin selection, cells were divided into three groups: 2 × 10^7^ cells without further treatment were harvested at −80 °C termed as DMSO_Day0_ group. The remaining cells were divided into another two groups, 2 × 10^7^ cells receiving 0.1% DMSO for another 14 days termed as DMSO_Day14_ group, and 2 × 10^8^ cells treated with 0.3 µM MMC for another 14 days termed as MMC_Day14_ group. After experiment stopped, cells of all the three groups were harvested for extracting genomic DNA. After two rounds PCR amplification, PCR products were purified and sequenced by Illumina 2500 by CloudHealth (Shanghai, China). The sequencing data were analyzed by both MAGeCK and gene score analysis^57^. CRISPR gene score (CS) = average [log_2_ (MMC_Day14_ sgRNA abundance/ DMSO_Day0_ sgRNA abundance)] or average [log_2_ (DMSO_Day14_ sgRNA abundance/ DMSO_Day0_ sgRNA abundance)]. PCR primers were shown in Supplemental Table 7.

### Statistical analysis

All statistical analyses were done in GraphPad Prism 7 software. Data were represented as Means ± SD with at least three replicates. Log-rank (Mantel-Cox) test was used for calculating *p* values of survival curves, One-way ANOVA using Tukey’s comparison test to compare multiple groups. Two-way ANOVA with Sidak’s multiple comparisons test or Dunnett’s multiple comparisons test were used where appropriate. Two-sided Fisher’s exact test was used to determine the association between *BRCA2* mutation and MSI or *TP53* mutation.

## Supplementary information

supplemental materials

Supplemental table 1

Supplemental table 2

Supplemental table 3-7

Supplemental Information summary

Supplementary Figure 1

Supplementary Figure 2

Supplementary Figure 3

## Data Availability

All the datasets are available from the corresponding author upon request. RNA sequencing data and GeCKO screening data have been deposited into NCBI database under accession number GSE132327 and GSE132431.

## Code availability

All the codes are publically available according to the references.
