# Supplementary material for: Brca2 deficiency drives gastrointestinal tumor formation and is selectively inhibited by mitomycin C
Source: Cell Death Dis. 2020 Sep 26;11(9):812. doi: 10.1038/s41419-020-03013-8 (PMC7519908; doi:10.1038/s41419-020-03013-8)
Supplement: Supplementary file 1 — supplemental materials [file 41419_2020_3013_MOESM1_ESM.docx]

**Supplemental materials for**

**Title: *Brca2* deficiency drives gastrointestinal tumor formation and is selectively inhibited by mitomycin C**

**Authors:** Xiaomin Chen^1^, Fangfei Peng^1^, Yan Ji^2^, Honggang Xiang^3^, Xiang Wang^1^, Tingting Liu^1^, Heng Wang^1^, Yumin Han^1^, Changxu Wang^1^, Yongfeng Zhang^1^, Xiangyin Kong^1^, Jing-Yu Lang^1,*^

**Affiliations:**

^1^CAS_Key Laboratory of Tissue Microenvironment and Tumor,

^2^Bioinformatics Core,

Shanghai Institute of Nutrition and Health, Shanghai Institutes for Biological Sciences, University of Chinese Academy of Sciences, Chinese Academy of Sciences, Shanghai, 200031, China.

^3^Department of General Surgery, Pudong New Area People’s Hospital affiliated to Shanghai University of Medicine & Health Science, Shanghai, 201299, China.

***Correspondence to:** Jing-Yu Lang, Biological Research Building A, 320 Yueyang Road, Shanghai, 200031, P. R. China. Phone: 86-21-54923285; E-mail: [jylang@sibs.ac.cn](mailto:jylang@sibs.ac.cn) (J.Y.L.).

**Running title:** BRCA2 is a crucial therapeutic target of Mitomycin C

**Keywords:** Gastrointestinal tumor, BRCA2, Mitomycin C, p53.

**Supplementary figure legends**

**Figure S1. Mutations of Fanconi anaemia pathway members in colorectal cancer and the genotyping of *Brca2* and *Trp53* conditional knockout mice.** (a) Mutation status of FA- and FA-like family members in colorectal adenocarcinoma samples (DFCI, Cell reports 2016). (b) Survival curves of colorectal cancer patients with BRCA2-low expression (n=373) and BRCA2-high expression (n=224). * p<0.05. (c) Schematic representation of the target alleles before and after Cre-mediated recombination of *Brca2* and *Trp53*. Exons are indicated as numbered boxes and *loxP* sites as triangles. Primers used for detecting the *loxP* sites are indicated as green arrows, the 5’ *loxP* site of *Brca2* (10F1, 10R1), the 3’ *loxP* site of *Brca2* (11F, 11R), and deletion of *Brca2* (10F1, 11R); the 5’ *loxP* site of *Trp53* (1F, 1R), the 3’ *loxP* site of *Trp53* (10F2, 10R2), and deletion of *Trp53* (1F, 10R2). PCR primer pairs were listed in supplemental table 3. (d) PCR analysis showing rearrangement of *Bcra2* alleles in response to *Villin-Cre* expression using DNA from the mouse tail, stomach and intestine. (e) PCR analysis showing rearrangement of *Bcra2* and *Trp53* alleles in response to *Villin-Cre* expression using DNA from the mouse tail, stomach and intestine. (f) H&E staining of stomach sections from *Villin-Cre^+^; Brca2^fl/fl^ ;Trp53^fl/+^* mice. Scale bar, 200 µm.

**Figure S2. *BRCA2* mutant gastrointestinal cancer cell lines are sensitive to DNA damaging agents, especially MMC.** (a) The mutation status of *BRCA1* and *BRCA2* in the indicated cancer cell line according to the CCLE database. (b) Wild-type, *BRCA1*-mutant and *BRCA2*-mutant cell lines were treated with 0.3 µM MMC or 0.1% DMSO for 72 hours, and cell viability was determined at OD570. (c) Wild-type, *BRCA1*-mutant and *BRCA2*-mutant cell lines were treated with 10 µM olaparib or 0.1% DMSO for 72 hours, and cell viability was determined at OD570. (d) Wild-type, *BRCA1-*mutant and *BRCA2*-mutant cell lines were treated with 10 µM melphalan or 0.1% DMSO for 72 hours, and cell viability was determined at OD570. (e) Wild-type, *BRCA1*-mutant and *BRCA2*-mutant cell lines were treated with 10 µM cisplatin or 0.1% DMSO for 72 hours, and cell viability was determined at OD570. (f) After treated with 1 µM or 10 µM olaparib for 5 days, the viability of HGC-27 cells was determined at OD570 with normalization to DMSO treatment. (g) After treated with 10 µM or 20 µM cisplatin for 9 days, the viability of HGC-27 cells was determined at OD570, with normalization to DMSO treatment. (h) Sanger sequencing data of the *BRCA2* mutant site in the SNU-1, SNU-5 and HGC-27 cell lines. (i) Sanger sequencing data of the *BRCA2* mutant site in sgBRCA2 (#1, #2) SNU-1 cells. Data represents as mean ± SD, * p < 0.05, ** p < 0.01, *** p < 0.01, ****p < 0.0001.

**Figure S3. Genome-wide knockout screen reveals that depletion of FA pathway members endow the sensitivity of gastrointestinal tumor cells to MMC.** (a) The BRCA2 protein expression level was determined in SNU-216 cells by immunoblotting after treatment with the indicated drugs for 24 hrs. (b) RT-qPCR analysis of BRCA2 mRNA expression levels in indicated cells in the presence of 3 μM MMC or 0.1% DMSO. (c) The BRCA2 protein and mRNA expression levels in SNU-216 cells were determined by RT-qPCR after treatment with the MMC for indicated time. (d) Using genome-wide CRISPR-Cas9 knockout screening, Five FA pathway members including FANCB, FANCF, FANCM, FANCP/SLX4 and FANCD2 were positively selected out in BRCA2 wild-type SNU-216 cells (p<0.05 with duplicated sgRNA hits), but they did not select out when BRCA2 is mutated. These positively selected FA pathway members were indicated by green color, showing decreased sgRNA levels on the left. Data represent the mean ± SD, * p < 0.05, ** p < 0.01, *** p < 0.01, ****p < 0.0001.

**Supplementary materials and methods**

**Compounds.** Mitomycin C was purchased from MedChemExprress (#HY-13316, Shanghai, China). Olaparib, Cisplatin and KU-55933 were purchased from Selleck Chemicals (Shanghai, China). N-Methyl-N-nitrosourea (MNU) and Melphan were purchased from Canspec China (Shanghai, China). 5FU and DMSO were purchased from Sigma-Aldrich (Saint Louis, USA). Thiazolyl blue tetrazolium bromide (MTT) was purchased from Amersco (#0793-1G, Shanghai, China).

**Plasmids.** Human GeCKOv2 CRISPR Knockout Pooled Library (#1000000048), LentiCRISPR v2 (#52961), pLX304 (#25890), pLX302 (#25896), pLKO.1-puro (#8453), pMD2.G (#12259), pRSV-Rev (#12253) and pMDLg/pRRE (#12251), pcDNA3 236HSC WT (BRCA2) (#16246) were purchased from Addgene.

**Antibodies.** Primary antibodies against Brca2 (OP95, 1:1000), phospho-H2A.X (Ser139) (#05363, 1:3000) and Noxa (OP180, 1:1000) were purchased from Millipore. Primary antibodies against cleaved PARP (#5625, 1:1000), phospho-p53 (Ser15) (16G8, 1:3000) were obtained from Cell Signaling Technology; Primary antibodies against Brca2 (sc-8326/sc-293185, 1:1000), β-tubulin (ab135209, 1:5000) were purchased from Abcam; Primary antibodies against Puma α/β (sc-28226, 1:2000), Vinculin (sc-25336, 1:3000), Rad51(H-92) (sc-8349, 1:200) and p53 (sc-126, 1:3000) were purchased from Santa Cruz Biotechnology.

**Immunochemistry staining.** When mice sacrificed, tumors at stomach and colon sites will be monitored and tumor tissues were isolated and fixed in 4% paraformaldehyde (PFA) (SHBH0586V, Sigma) for about 36 hours, and were then dehydrated and embedded in paraffin. Tumor sections were stained with hematoxylin and eosin (H&E) staining. Brca2 staining was performed on the formalin-fixed and paraffin-embedded tissue sections after antigen retrieval (Tris-EDTA, 10mM-1mM, pH 9). Tissues were incubated with an antibody recognizing BRCA2 protein (sc-8326, 1:200) at 4°C overnight, following with secondary antibody incubation at room temperature for 1 hour, and were then subjected for diaminobenzidine and hematoxylin staining. All images were captured by Zeiss Axio Imager A2 microscope.

**Mouse derived primary tumor cells.** Primary tumor cells were derived from *Villin-Cre^+^; Brca2^fl/fl^* mouse spontaneous tumors. Briefly, tumor tissues were cut into small pieces and were seeded into 10-cm plate that was pre-treated with FBS at 37°C, 5% CO2 for 10 min. Fresh DMEM/F-12 medium supplemented with 10% FBS and P/S was added and replaced every three days.

**Cell viability assay.** 3-5 x 10^3^ cells were seed into each well of 96-well-plate overnight before the treatment. After treated with indicated chemicals or vehicle (0.1% DMSO) for 72 hours, the cell viability was determined by MTT assay at OD570 nm.

**Trypan blue staining.** Tumor-derived primary cells were seeded in 96-well-plate at a density of 3000 cells per well. After adhered, cells were treated with indicated concentrations of MMC or 0.1% DMSO for 72 hours. 10 µL 0.4% Trypan blue solution was used to stain the dead cells.

**Cell cycle analysis.** After treated with 1 µM MMC or 0.1% DMSO for 24 hours, cells were harvested and fixed with 1 mL 70% ice-cold ethanol overnight at 4°C. Cells were stained with PI at 37°C for 30 min after PBS wash, and were analyzed using Beckman Gallios. Data were analyzed using FlowJo software.

***In vivo* tumor xenograft mouse model.** Tumor cells or tissues were subcutaneously transplanted into the right flank of immune-deficient mice. When tumor average volume is about 180 mm^3^, mice were divided into 3 groups (5 mice per group) and treated with vehicle (0.89% NaCl), MMC (3 mg/kg) or olaparib (50 mg/kg), once per week for total 3-4 weeks, respectively. Tumors were measured twice per week and was calculated by a formula: Tumor volume = 0.5× length × width^2^.

**Immunofluorescence staining.** For immunofluorescence using BRCA2 and Rad51 antibodies, cells were seeded onto cover slides in 24-well-plate and were then treated with 3 µM MMC for 18 hours after attached. After treatment stopped, cells were permeated with 0.2% TritonX-100 for 10 min and were then fixed with 4% paraformaldehyde for 15 min. After fixed, cells were incubated with 3% BSA at 4°C for another 1 hour in order to block the non-specific targets. Antibodies of γ-H2AX (#05363, 1:100),Rad51 (H-92) (sc-8349, 1:200), BRCA2 (OP95, 1:50), were prepared in 3% BSA and were incubated with cells overnight at 4°C. Alexa Fluor 488-conjugated secondary antibody (115-545-003/115-545-207, 1:200, Jackson ImmuoResearch) and Rhodamine Red-conjugated secondary antibody (115-295-205, 1:200, Jackson ImmuoResearch) were used to detect the immunofluorescence signal after incubated for another 1 hour at 25°C. DAPI was used to stain the nuclei. All images were captured using Zeiss Axio Imager A2 microscope. To quantify the positive percentage, 100-150 cells were analyzed.

**Immunoblotting.** Cells were lysed in a lysis buffer containing 50 mM Tris HCl (pH 8), 150 mM NaCl, 1% NP-40, 0.5% sodium deoxycholate, 0.1% SDS and 1x protease inhibitor cocktail (31880700, Roche). The protein concentration was quantified using Pierce BCA protein assay kit. 25 µg amounts of protein were loaded into each well of SDS-PAGE gels and were incubated with appropriate primary antibodies at 4°C overnight after blocked with 5% nonfat milk (EB27BA0032, BBI Life Sciences) or 5% BSA in TBST for 1 hour. Peroxidase-conjugated second antibodies were used to detect the signals after incubated for 1 hour at room temperature using an enhanced chemiluminescence reagent (1705041, Bio-Rad) on a Tanon 6100 machine.

**RNA-Seq.** 4 x 10^6^ cells were seeded into 10-cm-plate and treated with 3 µM MMC or vehicle (0.1% DMSO) for 36 hours after cells attached. Then the cells were harvested, washed with 1 x PBS for 3 times and lysed with TRIZOL reagent for total RNA extraction. The libraries were prepared, qualified by Novogen Company and sequenced on Illumina HiSeq X Ten platform. Sequenced pair-end reads were aligned into the human reference genome (GRCh38), and then were normalized to obtain TKM values using Salmon software.

**Comet assay.** 1 x 10^5^ cells were seeded into 6-well-plate and treated with 3 μM MMC for 36 hours. Cells were trypsinized, centrifuged and re-suspended in 1 x PBS. 25 μL cells were mixed with equal volume of 1% low melting agarose and were embedded in the slides which were pre-covered with 0.5% agarose, then solidified in 4°C for 15 min. Slides were subsequently incubated in 50 mL lysis solution (1% sodium N-lauroylasarcosinate, 10 mM Tris-HCl, 100 mM EDTA, and 2.5 M NaCl, pH 10, with the addition of 1% Triton X-100 and 10% DMSO, before use) for 90 min on ice with no light. After lysis, slides were washed and subsequently incubated with ice-cold alkaline denaturation buffer (300 mM NaOH and 1 mM EDTA, pH 13) for 20 min on ice with no light. For electrophoresis, slides were placed into electrophoresis chamber with ice-cold alkaline denaturation buffer for 20 min at 1.6 V/cm and at 4°C. After electrophoresis, slides were washed twice by neutralization buffer (0.4 M Tris-HCl, pH 7.5) for 5 min and 40 μL of DAPI was applied on each slide. Cells were observed using Zeiss Axio Imager A2 microscope.

**Real-time PCR.** Total mRNAs were extracted by TRIZOL reagent and was reversely transcribed into cDNA by PrimeScript RT reagent kit. Real-time PCR was performed on ABI-7500 PCR machine with NovoStart^®^SYBE qPCR SuperMix Plus. Primers used for real-time PCR were shown in supplemental table 4.

**CRISPR-Cas9 mediated knockout cells.** Cells were transiently transfected with pLenti-CRISPR-v2 containing indicated sgRNA using Lipofectamine® 3000. Non-targeting sgRNA and sgRNA targeting EGFP were used as internal control. After transfected for 48 hours, cells were replaced with fresh medium containing puromycin to remove the untransfected cells for another 72 hours. After puromycin selected, cells were seeded into 96-well-plate at a density of a single cell per well, and single cell subclones were harvested. Both immunoblotting and Sanger sequencing were used to validate the knockout efficacy of indicated genes. Primers were shown in supplemental tables 5 and 6.

**BRCA2 overexpression.** HGC-27 were transfected with pcDNA3-BRCA2 WT plasmid (Addgene #16246) and control vector using Lipofectamine® 3000, respectively. After 48 hours, cells were selected with neomycin (800 µg/mL) to remove un-transfected cells for another 3 days. After selection, cells were seeded into 96-well-plate at a density of a single cell per well with G418. After growth for 2 weeks, single cell subclones were harvested and validated by Sanger sequencing and were further subjected to MMC treatment. After treated with 3 µM MMC for 16 hours, respectively, the cell viability and transcript sequence of BRCA2-OE and control cells were determined. Primers used for Sanger sequencing were shown in supplemental table 6.
