# Supplementary figures and images for: Brca2 deficiency drives gastrointestinal tumor formation and is selectively inhibited by mitomycin C
Source: Cell Death Dis. 2020 Sep 26;11(9):812. doi: 10.1038/s41419-020-03013-8 (PMC7519908; doi:10.1038/s41419-020-03013-8)

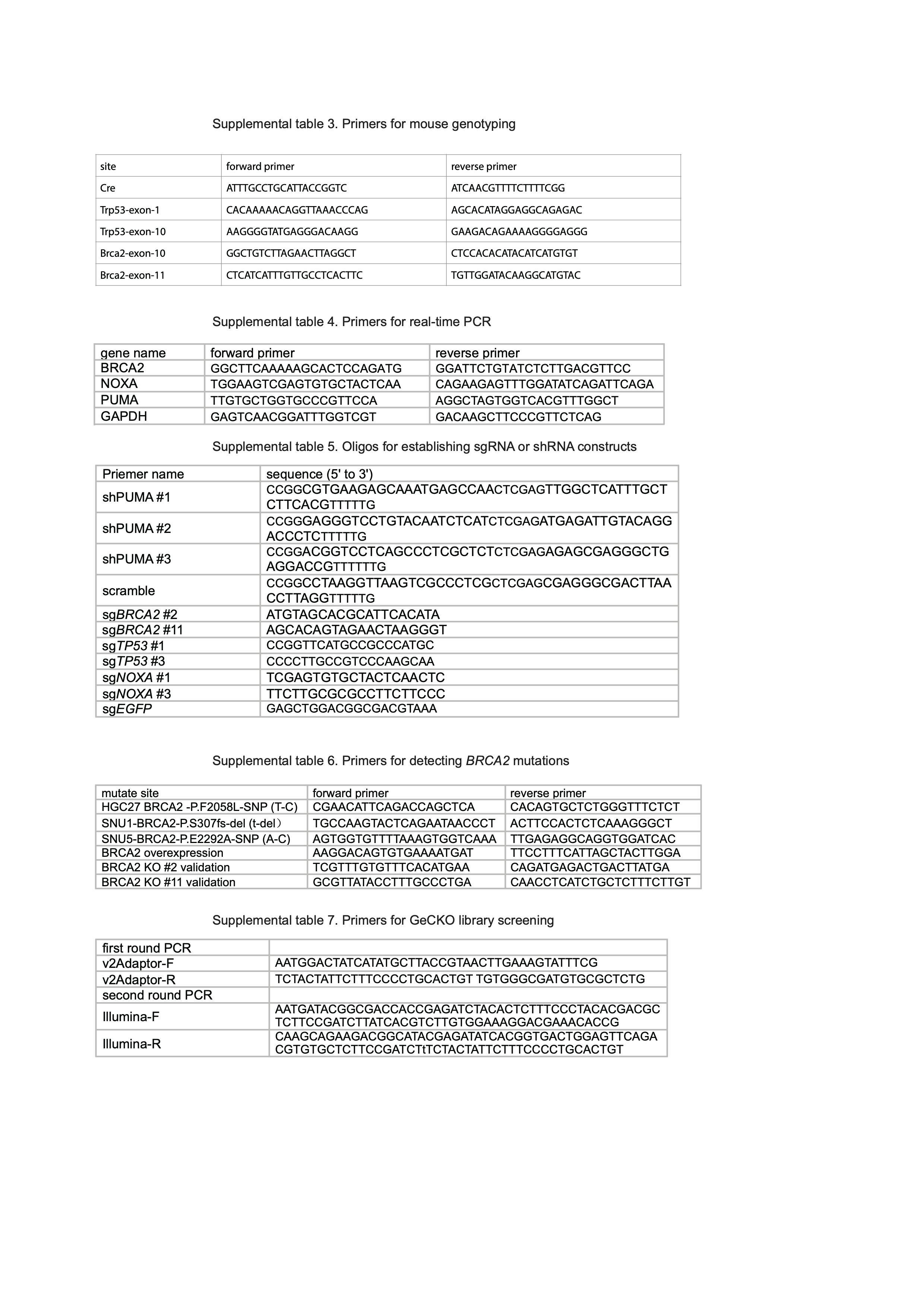

Supplement: Supplementary file 4 — Supplemental table 3-7 [file 41419_2020_3013_MOESM4_ESM.tif]

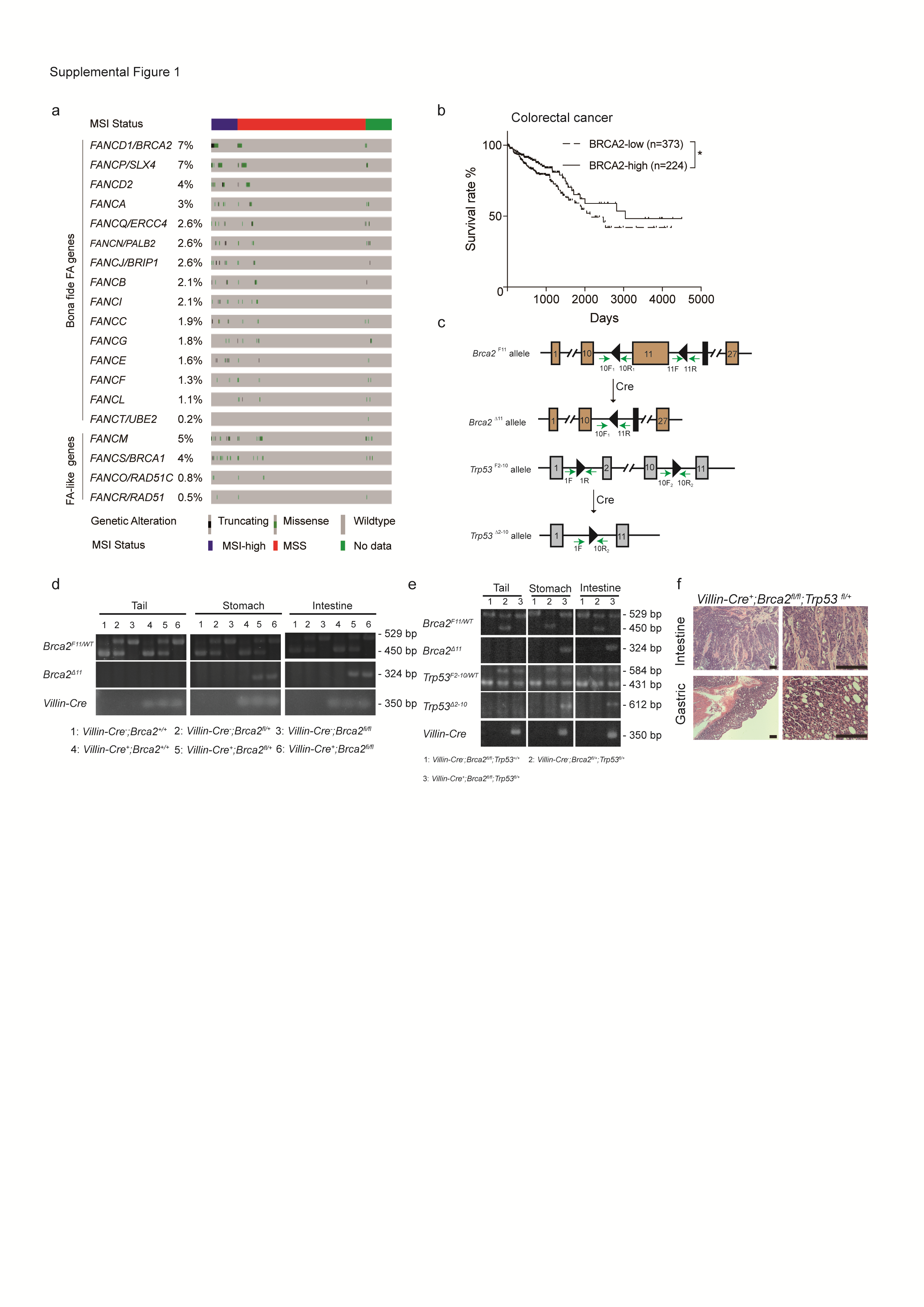

Supplement: Supplementary file 6 — Supplementary Figure 1 [file 41419_2020_3013_MOESM6_ESM.tif]

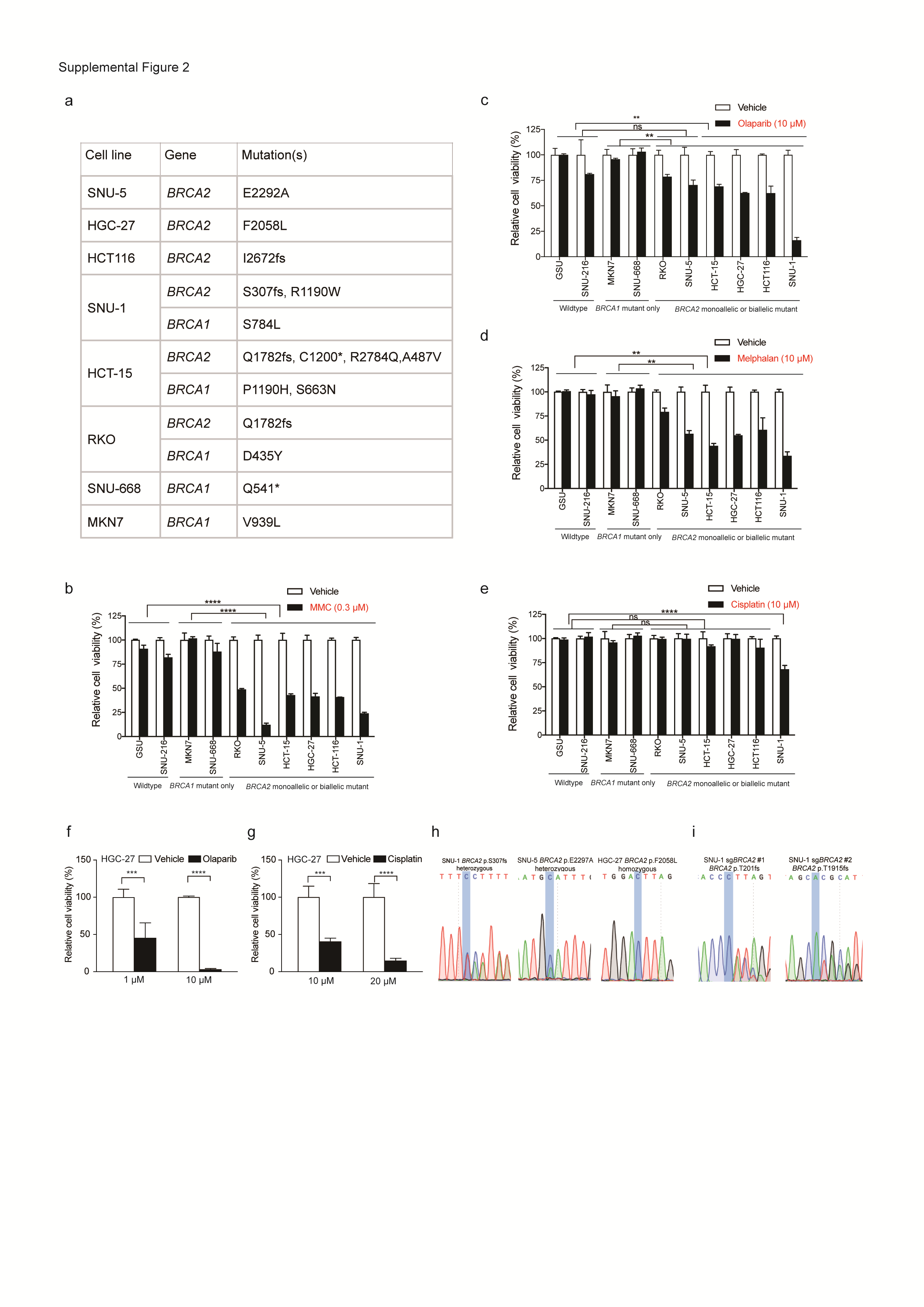

Supplement: Supplementary file 7 — Supplementary Figure 2 [file 41419_2020_3013_MOESM7_ESM.tif]

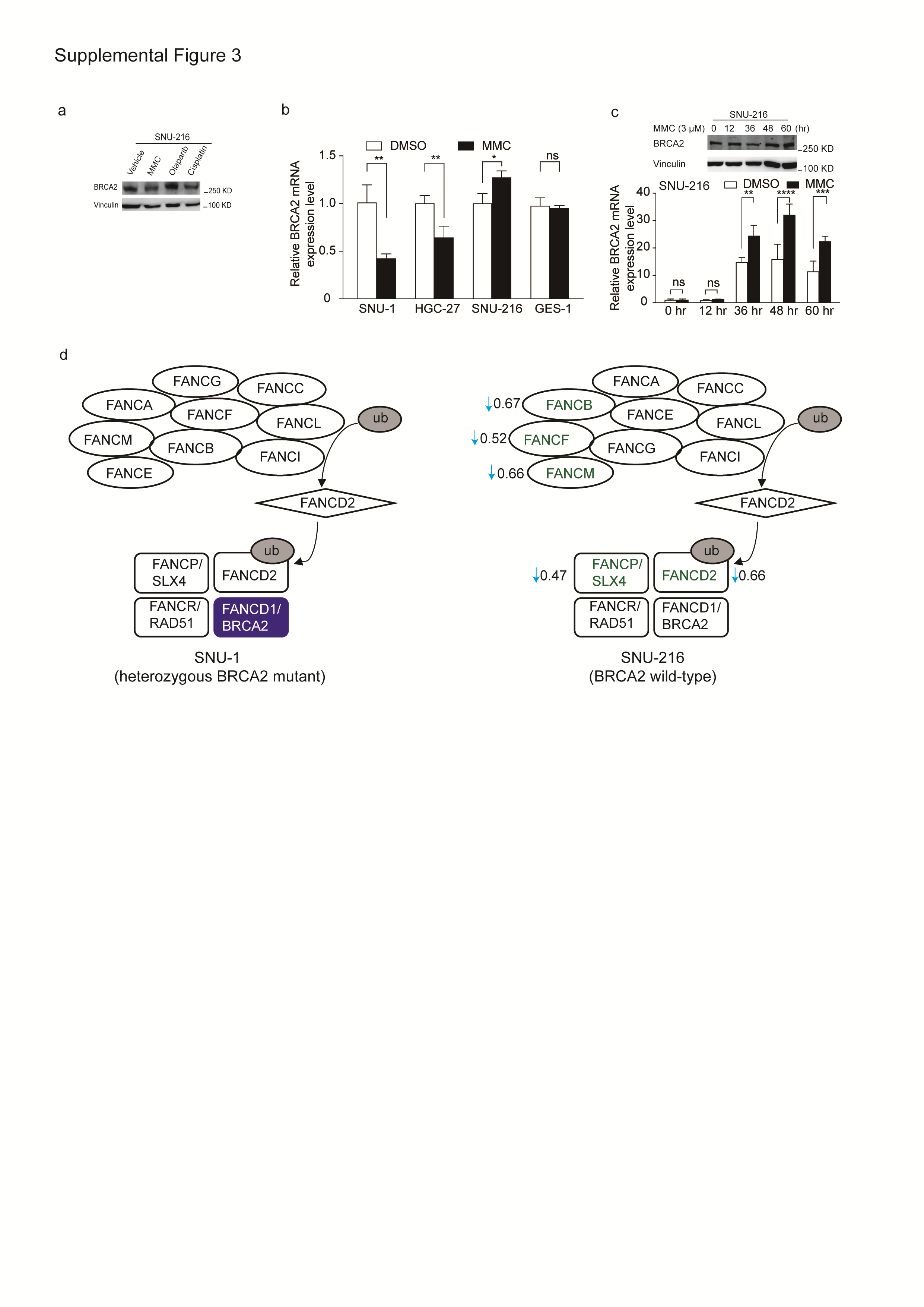

Supplement: Supplementary file 8 — Supplementary Figure 3 [file 41419_2020_3013_MOESM8_ESM.tif]
